# Post-ERCP pancreatogastric fistula associated with an intraductal papillary-mucinous neoplasm of the pancreas – a case report and literature review

**DOI:** 10.1186/1477-7819-3-70

**Published:** 2005-10-19

**Authors:** Masaru Koizumi, Naohiro Sata, Koji Yoshizawa, Munetoshi Tsukahara, Katsumi Kurihara, Yoshikazu Yasuda, Hideo Nagai

**Affiliations:** 1 Department of Surgery, Jichi Medical School, 3311-1, Yakushiji Minamikawachi Tochigi, 329-0498, Japan

## Abstract

**Background:**

Fistula formation has been reported in intraductal papillary-mucinous neoplasms (IPMNs) with or without invasion of the adjacent organs. The presence or absence of invasion is mostly determined by postoperative histological examination rather than by preoperative work-up.

**Case presentation:**

A 72 year-old Japanese woman showed remarkable dilatation of the main pancreatic duct (MPD) in the distal region of the pancreas. Subsequent ERCP also showed MPD dilatation, after which the patient suffered moderate pancreatitis. A subsequent gastroscopy revealed a small ulceration that had not been observed in a gastroscopy performed 3 months prior. Mucinous discharge from the ulceration suggested it might be the orifice of a fistula connected to the MPD. *En bloc *resection including the distal region of the pancreas, spleen, stomach and part of the transverse colon was performed under the pre- and intraoperative diagnosis of an invasive malignant IPMN. However, histopathology revealed the lesion to be of "borderline malignancy" without apparent invasion of the stomach. Light microscopy showed inflammatory cellular infiltrates (mainly neutrophils) around the pancreatogastric fistula, but there was no evidence of neoplastic epithelia lining the fistulous tract.

**Conclusion:**

This case highlights that a pancreatogastric fistula can develop after acute inflammation of the pancreas in the absence of cancer invasion. Further information regarding IPMN-associated fistulae is necessary to clarify the pathogenesis, diagnosis, appropriate surgical intervention and prognosis for this disorder.

## Background

Intraductal papillary-mucinous neoplasms (IPMNs) of the pancreas have unique clinico-pathological characteristics and show a wide spectrum of histological types, ranging from adenomatous hyperplasia to invasive cancer. While non-invasive IPMNs show slow growth and good prognosis after resection, the outcome can become poor if they transform into invasive ductal cancers. There are three types of IPMN: main duct,, branch, and combined. The main duct and combined types are associated with higher rates of invasive cancer than the branch type [1].

A feature of IPMNs is occasional fistula formation with surrounding organs such as the duodenum, bile ducts, stomach and even the peritoneal or pleural cavity [2-10]. While such fistula formation is generally thought to be associated with invasive IPMNs, some non-invasive IPMNs also form fistulas. Thus, IPMN fistula formation raises intriguing issues concerning pathogenesis, diagnosis and patient prognosis.

We report here a rare non-invasive "borderline-malignant" IPMN that formed a pancreatogastric fistula. Fistula formation was triggered by acute pancreatitis following endoscopic retrograde cholangiopancreatography (ERCP).

## Case presentation

**Figure 1:**
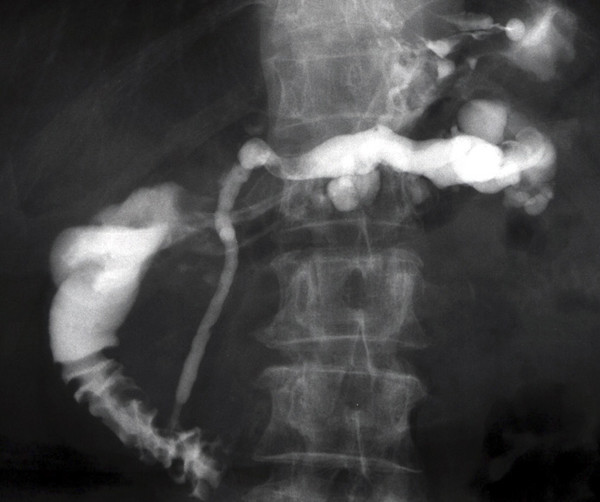
ERCP on presentation showing a dilated MPD and cystic lesions in the distal region of the pancreas.

A 72 year-old Japanese woman underwent abdominal ultrasonography (US) as part of a routine physical checkup in April 2001. This procedure incidentally detected marked dilatation of the main pancreatic duct (MPD) in the distal region of the pancreas. Magnetic resonance cholangiopancreatography (MRCP) and abdominal computed tomography (CT) showed MPD dilatation and a multi-lobular cystic lesion. ERCP confirmed these findings, and no extraluminal leakage of the contrast medium was observed (Figure 1). Cytology on pancreatic fluid indicated non-neoplastic epithelia. Endoscopic ultrasonography (EUS) identified 8 mm papillary projections in the cystic lesion, which was a key indication for surgery. A routine gastroscopy performed simultaneously with EUS showed no significant findings.

**Figure 2:**
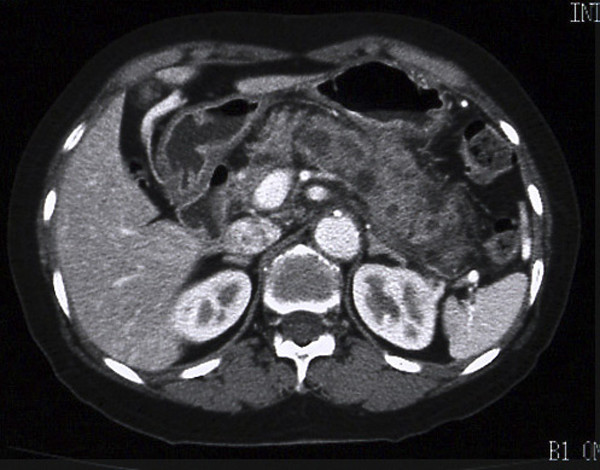
Abdominal CT just prior to surgery showing distinct swelling of the pancreas and the gastric wall, causing the border between the two organs to be unclear.

**Figure 3:**
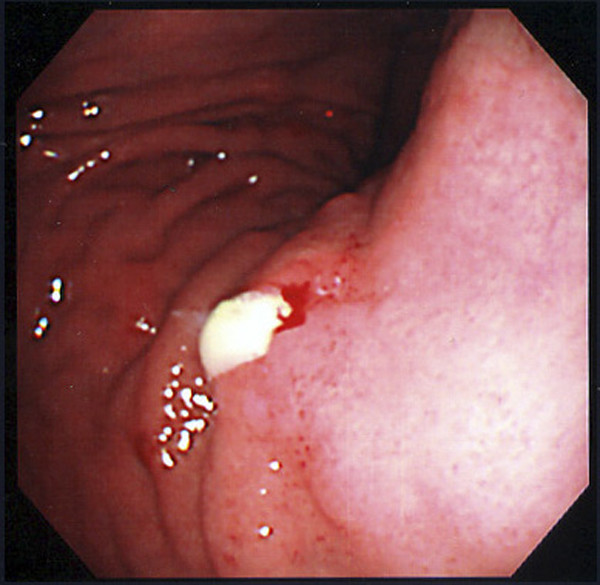
Gastroscopy just prior to surgery showing a small ulcer producing a mucinous discharge into the body of the stomach.

Following the ERCP, the patient complained of abdominal pain and showed hyperamylasemia and leukocytosis. Distinct swelling of the pancreas and thickening of the gastric wall were subsequently observed by abdominal CT (Figure 2), which indicated a mild post-ERCP pancreatitis. Gastroscopy performed for the assessment of gastric wall thickness revealed a small ulceration in the body of the stomach (Figure 3), which was accompanied by a mucinous discharge suggesting the presence of a fistula opening into the MPD. These findings, along with abdominal angiography data indicating encasement of the splenic artery suggested a malignant invasion into the stomach by the primary lesion. All other laboratory data, including tumor marker expression (e.g., CEA and CA19-9), were within normal limits.

**Figure 4:**
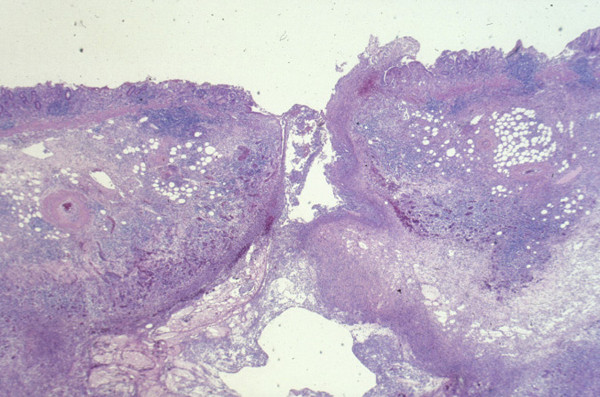
A cross-section of the pancreatogastric fistula (Hematoxylin and Eosin stain × 40). The fistula was significantly infiltrated with neutrophils and showed no evidence of neoplastic infiltration.

A laparotomy was performed 1 month after the ERCP. Firm fibrous tissue between the pancreas and the mesenterium of the transverse colon suggested the pancreatic neoplasm was of an invasive nature. An *en bloc *resection of the distal pancreas, spleen, stomach and part of the transverse colon was performed. Postoperative histopathology showed the distal pancreas had an IPMN whose lining epithelia showed only "borderline-malignancy" and no apparent invasion of adjacent tissues or organs. Light microscopy examination revealed inflammatory cellular infiltration consisting mainly of neutrophils around a pancreatogastric fistula which had no neoplastic epithelia (Figure 4). The patient was discharged after an uneventful postoperative recovery and showed no signs of recurrence 3 years after surgery.

## Discussion

Fistula formation associated with an IPMN was first reported by Ohhashi *et al *in 1980 as a pancreatobiliary fistula [2]. Since then there have been 41 cases reported between 1980 and 2004 (Table 1) [3-9]. According to these reports, the organs most frequently affected by fistula formation were the duodenum (24 cases, 59%), common bile duct (21 cases, 51%) and stomach (7 cases, 17%). Apparent cancer invasion was the main cause of the fistulae in 16 cases (37%), while similar to the present case, 17 cases (41%) showed fistulae into neighboring organs in the absence of tumor invasion (Table 2). Five of the six previous IPMN cases associated with pancreatogastric fistulae were linked to apparent cancer invasion.

**Table 1:** Published cases of developing fistulae from IPMNs (1980–2004)

**No.**	**Author**	**Year**	**Affected Organs**	**Cancer Invasion**	**Pathological Diagnosis**
1	Ohhashi [2]	1980	C	-	cystadenocarcinoma
2	Koike [3]	1980	C, D	unknown	pap
3	Sukisaki [3]	1984	S, D	+	pap (+ muc)
4	Usui [3]	1985	D	-	pap
5	Suyama [3]	1986	C	+	pap
6	Suyama [3]	1986	D	+	pap
7	Yamao [3]	1986	D	unknown	pap
8	Yamao [3]	1986	C	unknown	pap
9	Yamao [3]	1986	C	unknown	pap
10	Miyagawa [3]	1988	C, D	+	pap (+ muc)
11	Kuga [3]	1988	D	-	pap
12	Tohyama [3]	1989	C	-	pap
13	Nakajima [3]	1989	C	-	pap
14	Ohnuma [3]	1990	S	+	pap (+ muc)
15	Sakimoto [3]	1991	C	+	pap (+ muc)
16	Mayumi [3]	1991	C, D	+	muc (+ pap)
17	Hayashi [3]	1991	C	unknown	cystadenocarcinoma
18	Oda [3]	1993	C, D	-	pap
19	Saitoh [3]	1993	C	-	pap
20	Kobayashi [4]	1993	S	+	muc (+ pap)
21	Kobayashi [4]	1993	S, D	+	muc (+ pap)
22	Kobayashi [4]	1993	C, D	unknown	pap
23	Kobayashi [4]	1993	D	unknown	pap
24	Kobayashi [4]	1993	D	unknown	muc (+ pap)
25	Mori [3]	1994	C	+	pap
26	Ieda [3]	1995	D	+	tub
27	Yago [5]	1995	S	-	pap
28	Takekuma [3]	1996	D	-	pap
29	Nakamura [3]	1996	C, D	+	pap
30	Murata [3]	1996	C, D	-	pap
31	Hirota [3]	1996	D	-	pap
32	Tadokoro [6]	1996	D	-	unknown
33	Nakatsuka [3]	1997	S, D	+	muc
34	Matsubayashi [7]	1998	D	+	pap
35	Kawaharada [3]	1999	D	+	pap
36	Kawaharada [3]	1999	D	+	pap
37	Shiroko [8]	2000	C, D	-	unknown
38	Kurihara [9]	2000	C	-	pap
39	Kurihara [9]	2000	C	-	pap
40	Fujisawa [3]	2001	C	-	pap
41	Koizumi(present case)	2004	S	-	low grade malignancy

Affected Organ; C: Common bile duct, D: Duodenum, S: Stomach

Pathological Diagnosis; pap: papillary carcinoma, muc: mucinous carcinoma

tub: tubular adenocarcinoma

**Table 2:** Organs developing IPMN-associated fistulae (Revised from Table 1)

		**Cancer invasion**		
		
**Affected organs**	**Case**	**+**	**-**	**unknown**
C	13	3	7	3
D	13	5	5	3
C and D	8	3	3	2
S	4	2	2 (this case)	0
S and D	3	3	0	0
Total	41	16	17	8

Affected Organ; C: Common bile duct, D: Duodenum, S: Stomach

To our knowledge, the present report is only the second describing a case of IPMN associated with pancreatogastric fistulae without invasive cancer. Yago *et al *reported a rare case of non-invasive intraductal papillary adenocarcinoma of the pancreas that was associated with development of a pancreatogastric fistula in the absence of cancer invasion [5]. In that case, neoplastic epithelia were observed only on the surface of the fistula and gastric mucosa, while the structure of the gastric wall beside the fistula was maintained without invasive cancer. Those authors speculated that the pancreatogastric fistula developed due to high pressure in the MPD. Baek *et al *and Watanabe *et al *reported the same phenomenon in mucinous cystic tumors (MCTs) [11,12]. The mechanism of such fistula formation without cancer spread could be explained by a combination of high pressure in the MPD and inflammatory stimulation [3,4,13].

In the present case, ERCP played a key role in fistula formation. To our knowledge, this is the first report to show an absence of a pancreatogastric fistula immediately prior to ERCP, and then the presence of a fistula following post-ERCP pancreatitis. Post-ERCP pancreatitis, which is reported to occur in approximately 1.8–10% of patients, should be carefully investigated in IPMN cases [14,15].

We emphasize here that development of an IPMN-associated fistula does not necessarily represent underlying invasive cancer. Fistula formation can result in an incorrect preoperative diagnosis of invasive cancer and lead to the undertaking of unnecessary surgical procedures. Although the lesion in the present case was a non-invasive tumor, surgery was appropriate given the indications of invasive cancer. While non-invasive IPMNs have a good prognosis, there remains the possibility of dissemination by tumor penetration. When treating IPMNs associated with fistula, the extent of resection should depend on the extent of cancer invasion. To avoid dissemination, the fistula should be removed independent to cancer invasion. The extent of resection of affected organs should be individually determined based on the results of preoperative images and precise intraoperative histological examinations.

## Conclusion

The present case highlights that a pancreatogastric fistula can develop in the absence of invasive cancer. Further data regarding IPMN-associated fistulae are necessary in order to clarify the pathogenesis, diagnosis, appropriate surgical intervention and prognosis for this disorder.

## Competing interests

The author(s) declare that they have no competing interests.

## Authors' contributions

MK, NS, KY, MT, KK, YY and HN were involved in performing surgery, undertook the literature search and drafted the manuscript for submission.

HN supervised preparation of the manuscript and edited the final version for publication. All authors read and approved the manuscript.
